# Synthesis, Characterization, DNA Binding, and Photocleavage Activity of Oxorhenium (V) Complexes with *α*-Diimine and Quinoxaline Ligands

**DOI:** 10.1155/2010/973742

**Published:** 2010-06-08

**Authors:** Christiana A. Mitsopoulou, Constantinos Dagas

**Affiliations:** Laboratory of Inorganic Chemistry, Department of Chemistry, University of Athens, Panepistimiopolis, Zografou, Athens 157 71, Greece

## Abstract

The complex [ReOCl_3_pq] (**1**) (where pq = 2-(2′pyridyl)quinoxaline) has been synthesized and fully characterized by UV-Vis, FTIR, 1 and 2D NMR, and cyclic voltammetry (CV). The DNA-binding properties of the complex **1** as well as of the compounds [ReOCl_3_bpy] (**2**), [ReOCl_3_phen] (**3**), and pq (**4**) were investigated by UV-spectrophotometric (melting curves), CV (cyclic voltammetry), and viscosity measurements. Experimental data suggest that complex **1** intercalates into the DNA base pairs. Upon irradiation, complex **1** was found to promote the cleavage of plasmid pBR 322 DNA from supercoiled form I to nicked form II. The mechanism of the DNA cleavage by complex **1** was also investigated.

## 1. Introduction

For many years transition metal complexes have piqued curiosity owing to their efficient DNA binding and cleavage properties under physiological conditions [1–14]. It has been demonstrated that inorganic complexes can be used in footprinting studies, as sequence specific DNA binding agents, as diagnostic agents in medicinal applications, and for genomic research. Among different modes of DNA cleavage, oxidative cleavage of DNA upon irradiation with visible light is of main interest due to the potential applications of such compounds in photodynamic therapy of cancer [3, 15] and references therein.

On the other hand, coordination chemistry of rhenium has been extensively developed in recent years due, to a large extent, to the fact that its complexes with diimine ligands display long lifetimes and also short-lived rhenium isotopes hold promise as *β*-emitters in radiotherapy [14, 16, 17]. The chemistry of oxorhenium complexes arouses particular interest among these compounds not only for their implication in various reactions of industrial and biological importance, including olefin epoxidation and catalysis by cytochrome P-450 [18, 19], but also for their lipophilic character and the oxidation states of rhenium that is Re(I) to Re (VI) [17].

In this context, the design, synthesis, and reactivity of novel rhenium oxocomplexes have become the aim of several laboratories, including ours. To the best of our knowledge, studies on oxorhenium (V) complexes incorporating planar aromatic ligands that could bind to DNA have not been studied before. Nevertheless, quinoxaline (pq) has recently received considerable attention [20, 21]. The structure of the quinoxaline ligand is recognized from a great number of natural compounds such as riboflavin and molybdopterines, and can be used as antibacterial, antiviral, anticancer, antihelmintic, and insecticidal agent [22]. In addition, it adopts a planar conformation when chelates to a metal ion [20, 23, 24]. This planar appendage provides a handle for intercalative binding to DNA, positioning the complex for enhanced reactivity toward DNA.

**Figure 1:**
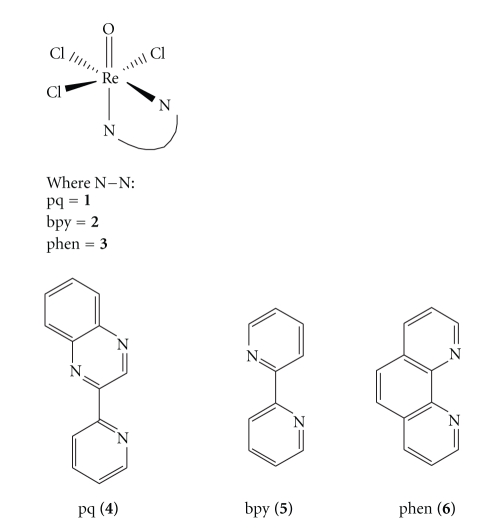
Structures of the complexes **1**–**3** and the corresponding *α*-diimine ligands.

In this paper, we present the synthesis and characterization of the oxorhenium (V) complex, [ReOCl_3_pq] **(1)**. In order to elucidate the reactivity of this compound, we have also synthesized the complexes [ReOCl_3_bpy] **(2)** and [ReOCl_3_phen] **(3) **and studied their intercalating abilities. The ligands (2′pyridyl)quinoxaline **(4)**, 2,2′ bipyridine **(5)**, and 1-10-phenanthroline **(6)** belong to quinoxaline or diimine class of organic compounds (Figure 1). 

The interaction of these compounds with double stranded calf thymus DNA has been investigated using circular dichroism spectroscopy (CD), DNA thermal denaturation analysis (melting point), CV, and viscosity measurements. The photocleavage reaction on DNA has been monitored by agarose gel electrophoresis. Understanding the interactions between the compounds under study and DNA, and their ability to damage DNA with photoactivation by visible light would be the first step toward the development of rhenium-based drugs which might be useful in photobiological applications such as photodynamic therapy.

## 2. Experimental

### 2.1. Materials

2-(2′-pyridyl)quinoxaline [25, 26] and [ReOCl_3_(PPh_3_)_2_] [27] were prepared by reported procedures. All reactions and manipulations were conducted under a pure argon atmosphere using standard Schlenk techniques. Ultra-pure Milli-Q water (18.2 mΩ) was used in all experiments. Reagent grade solvents were dried and distilled by usual methods and the solvents were stored over molecular sieves (4$\dot{\text{A}}$). The chemicals were purchased by Aldrich and used as received. Calf thymus DNA (CT DNA) and pBR322 supercoiled plasmid DNA (stored at: −20°C) were purchased from Sigma (St. Louis, MO).

### 2.2. Methods and Instrumentation

Microanalysis (C, H, N) was carried out with a Euro Vector EA 3000 analyser. FT-IR spectra in solution and in KBr pellets were recorded on a Nicolet Magna IR 560 spectrophotometer with a 1.0 cm^−1^ resolution. UV-Vis spectroscopy was recorded on a Varian Cary 300E spectrophotometer at 25 ± 0.2°C using cuvettes of 1 cm path length. The ^1^H NMR spectra were obtained at room temperature using a Varian Unity Plus 300 MHz spectrometer. Samples were run in a 5 mm probe with deuterated solvents as internal lock and reference. The assignment of the ^1^H NMR spectra of the free ligands and of the complexes is based on 2D NMR experiments (^1^H–^1^H COSY). A Fisons VG Quattro instrument with a VG Biotech Electrospray source, having a hexapole lens was employed for ESI-MS analysis [28].

Photolysis experiments were carried out with a 1000 W Xenon lamp in an Oriel, model 68820, Universal Arc. Lamp source was selected with appropriate interference filter (Corning).

Cyclic voltammetric (CV) measurements were performed in a single compartment cell with a three electrode configuration on a Pine AFCBP1 (pine Instrument Company, Groove City, PA, USA). Glassy carbon was the working electrode, and the reference electrode was a saturated calomel electrode (SCE). A platinum wire was used as the counter electrode. 

All the experiments involving interaction of the complexes with DNA were conducted in twice distilled phosphate buffer (Titrisol, 5 mM) and NaCl (4 mM) and adjusted to pH = 7.00 or 5.00 with hydrochloric acid. A solution of calf thymus DNA (CT-DNA) in the buffer gave a ratio of UV absorbance at 260 and 280 nm of ca. 1.8-1.9 : 1, indicating that the DNA was sufficiently free of protein [29]. The concentration of DNA in nucleotide phosphate was determined by UV absorbance at 260 nm after dilution. The extinction coefficient, *ε*_260_ was taken as 6600 M^−1^ cm^−1^ [29]. Stock solutions were stored at 4°C and used after no more than 4 d. Supercoiled plasmid pBR322 DNA was stored at −20°C and its concentration in base pairs was determined by UV absorbance at 260 nm after appropriate dilutions taking *ε*_260_ as 13 100 M^−1^ cm^−1^.

### 2.3. DNA Binding Studies

Thermal denaturation experiments were performed with a Varian Cary 300 spectrophotometer. Samples for Tm measurements were obtained by adding 100 *μ*L of a freshly prepared stock solution of rhenium compound, dissolved in the buffer (4 Mm NaCl, 0.096 M KH_2_PO_4_, 0.2 M Na_2_HPO_4_, pH 5.0 or 4 mM NaCl, 0.026 M KH_2_PO_4_, 0.041 M Na_2_HPO_4_, pH 7.0), to 1.00 mL of a calf thymus DNA solution (60 *μ*g/mL) and incubating at 25°C for 24 hours. As the solubility of compound **1** is poor in net water, it was firstly dissolved in MeOH (in any case the ratio of MeOH : water is no higher than 5 : 95). Under these circumstances, complex **1** is stable for at least a period of five days. Absorbance versus temperature profiles of DNA were measured at 258 nm in the temperature range from 25°C to 95°C. Temperature was raised in 0.5°C increments, and DNA complex samples were allowed to equilibrate for 1 minute at each temperature. The melting temperature (*T*_*m*_) of DNA was determined as the middle point of the hyperchromic transition. To correct the absorbance spectrum of DNA from the contribution of each of the studied compounds, the buffer solution of the corresponding complex at the same concentration in the sample was used as a blank. 

Viscosity measurements were carried out using a Schott Geräte AVS 310 viscometer maintained at a constant temperature at 25.0 (±0.1)°C in a thermostatic bath. DNA samples with approximately 200 base pairs in average length were prepared by sonication in order to minimize complexities arising from DNA flexibility. Flow time was measured with a digital stopwatch, while each sample was measured three times, and an average flow time was calculated. Data are presented as (*η*/*η*_0_)^1/3^ versus 1/*r*, where *r* = [DNA]/[complex **1**], *η* is the viscosity of DNA in the presence of complex, and *η*_0_ is the relative viscosity of DNA alone. The relative viscosity of DNA in the presence and absence of the metal complex was calculated using the expression *η* = (*t* − *t*_0_)/*t*_0_, where *t* is the observed flow time of the DNA solution and *t*_0_ the flow rate of buffer alone. According to Cohen and Eisenberg [30] the relationship between the relative solution viscosity (*η*/*η*_0_) and contour length (*L*/*L*_0_) is given by the equation *L*/*L*_0_ = (*η*/*η*_0_)^1/3^, where *L*_0_ denotes the apparent molecular length in the absence of the metal complex.

### 2.4. DNA Photocleavage

For the gel electrophoresis experiment, pBR322 supercoiled plasmid DNA (0.1 lg) was treated with the Re (V) complexes in 50 mM Tris-acetate, 18 mM NaCl buffer (pH 7.2), and the solution was then irradiated at room temperature with a light >400 nm (100 W) inside a photoreactor. The samples were analyzed by electrophoresis for 1 hour at 100 V in Tris-acetate buffer containing 1% agarose gel. The gels were imaged with a BioSure UV-Transilluminator and photographed using a Picture Works Photo Enhancer v3.2 digital camera equipped with a 10 *μ*g/mL ethidium bromide filter.

### 2.5. Synthesis of Complexes

In this context, rhenium (V) metal complexes with bidentate (N,N) type ligands as 2-(2′pyridyl)quinoxaline (pq) **4**, 2,2′-bipyridine (bpy) **5**, and 1,10-phenanthroline (phen) **6**, have been amply studied.

Ligands **5 **and **6** were purchased by Aldrich and used as received, whereas ligand **4** was synthesized according to published procedures [26, 31].

#### 2.5.1. Synthesis of [ReOC*l*_3_pq] (1)

Potassium perhenate (KReO_4_) was used as a starting material. A solution of triphenylphosphine (PPh_3_) (5.15 g, 18 mmol) in hot ethanol (29 mL) was added in a boiling mixture of potassium perhenate (1 g, 3.4 mmol), hydrochloric acid 37% (5.72 mL), and ethanol (5.72 mL). A yellow solid precipitates. The mixture was refluxed for 30 minutes and after cooling to room temperature, the yellow precipitate was filtered off, washed with ethanol, and dried *in vacuo*. By this procedure the precursor [ReOCl_3_(PPh_3_)_2_] was obtained. The ligand pq was added in a solution of [ReOCl_3_(PPh_3_)_2_] in dry methanol (20 mL) under stirring, and the mixture was heated to continuous reflux for 24 hours at 50°C. After cooling to room temperature, the resultant dark purple precipitate was filtered off, washed with diethylether, and dried *in vacuo*. 

Yield: 57%. (0.732 g) ^1^H NMR (300 MHz; CD_3_OD; s, singlet; d, doublet; t, triplet; m, multiplet): *δ* 9.73(s, 1H, H_3pq_), 8.15, 8.25(mt, 2H, H_7−10pq_), 7.92(mt, 2H, H_8-9pq_), 7.48(d, 1H, H_16pq_, *J* = 8.0), 8.08(mt, 1H, H_15pq_), 8.91(mt, 1H, H_14pq_), 8.65(d, 1H, H_13pq_, *J* = 5.5). Absorption spectrum: *λ*_max _(MeOH)  = 571.0 nm (*ε* = 3330 M^−1^ cm^−1^), *λ*_max _(MeOH)  =   732.1 nm (*ε* = 1227 M^−1^ cm^−1^). FT-IR spectrum: 939(vs) cm^−1^ (*ν*Re=O), 1630(m,br), 1580(w) and 1420(m) cm^−1^ (*ν*C–N) and (*ν*C=C). Anal. Calcd for ReOCl_3_pq: C 30.27; H 1.76; N 8.15; Cl 20.62; O 3.10. Found: C 30.97; H 1.74; N 8.19%. ES-MS (CH_3_CN: m/z (M—H^+^) 516.8.

#### 2.5.2. Synthesis of [ReOC*l*_3_bpy] (2)

This compound was prepared by refluxing [ReOCl_3_(PPh_3_)_2_] (0.83 g, 1 mmol) and bpy (1.3 mmol) in methanol (50 mL) for 6 hours using the procedure employed for **1**. A red microcrystalline material was isolated.

Yield: 88%. (0.554 g) ^1^H NMR (300 MHz; CD_3_COCD_3_; s, singlet; d, doublet; t, triplet; m, multiplet): *δ* 9.09(d, 2H, H_6−9bpy_, *J* = 7.9), 8.03(d, 2H, H_12−13bpy_, *J* = 5.9), 7.84(t, 2H, H_4−11bpy_), 7.37(t, 2H, H_5−10bpy_). Absorption spectrum: *λ*_max _(MeOH) = 480.8 nm (*ε* = 3430 M^−1^ cm^−1^), *λ*_max _(MeOH) = 750.2 nm (*ε* = 1450 M^−1^ cm^−1^), FT-IR spectrum: 988 cm^−1^ (*ν*Re=O), 1640–1570 cm^−1^ (*ν*C=C, (bpy)). Anal. Calcd for ReOCl_3_bpy: C 25.84; H 1.74; N 6.03; Cl 22.89; O 3.44. Found: C 26.03; H 1.79; N 6.19%. ES-MS (CH_3_CN: m/z (M—Cl^+^) 465.9.

#### 2.5.3. Synthesis of [ReOC*l*_3_phen] (3)

This compound was prepared by refluxing ReOCl_3_(PPh_3_)_2_ (0.83 g, 1 mmol) and phen (0.26 g 1.3 mmol) in methanol (50 mL) for 48 hours using the procedure employed for **1**. A dark red microcrystalline material was isolated. 

Yield: 84%. (0.843 g) ^1^H NMR (300 MHz; CD_3_COCD_3_; s, singlet; d, doublet; t, triplet; m, multiplet): *δ* 8.90(d, 2H, H_2-9phen_, *J* = 6.9), 8.37(d, 2H, H_4-11phen_), 7.40(s, H_phen_), 7.69(mt, H_phen_). Absorption spectrum: *λ*_max _(MeOH) = 459.6 nm (*ε* = 3200 M^−1^ cm^−1^), *λ*_max _(MeOH) = 739.6 nm (*ε* = 1100 M^−1^ cm^−1^), FT-IR spectrum: 985 cm^−1^ (*ν*Re=O), 1628(w), 1605(m), 1575(w), 1520(m) cm^−1^ (*ν*CN and *ν*C=C, (phen)). Anal. Calcd for ReOCl_3_phen: C 29.37; H 2.05; N 5.71; Cl 21.67; O 3.26. Found: C 30.03; H 2.17; N 5.99%. ES-MS (CH_3_CN: m/z (M—Cl^+^) 489.8.

## 3. Results and Discussion

### 3.1. Synthesis

2-(2′pyridyl)quinoxaline (pq) is produced via an unusual condensation reaction from 2-acetylpyridine and 1,2-diaminobenzene and has been extensively studied because of its rich coordination chemistry. 2-(2′pyridyl)quinoxaline belongs to the general class of quinoxalines which are natural products, used as antibiotics and form polymers with peculiar magnetic and electric properties. Their significant redox chemistry and photochemistry are responsible for many considerable intra and intermolecular electron transfer organic and biochemical processes. The aforementioned ligand as well as bpy and phen has been coordinated to the oxorhenium moiety. 

The most significant m/z peaks in ESI-mass spectra of complexes **1**–**3** are given in the Synthesis Section. They correspond to the molecular weight of the complex increased by one unit for complex **1**, so that [M-H^+^] form is attributed, implying that a proton has been attached on the N(3) atom of the quinoxaline ring. On the other hand, complexes **2** and **3** eliminate a -Cl atom providing the form [M-Cl]^+^. Surprisingly, we were not able to isolate crystalline complex from the oxorhenium-quinoxaline system in which only the typical chelate bonding of an *α*-diimine ring is present. In fact, up to now only few oxorhenium-*α*-diimine crystal structures have appeared in the literature though it is well-known that radioactive ^186^Re and ^188^Re are important diagnostic nuclear agents. Actually, the crystal structure of the ReOCl_3_phen was recently reported [32]. According to the data presented therein, the rhenium atom is in a distorted octahedral environment with the three chloride ligands arranged in a meridional fashion and the oxo ligand *trans* to the nitrogen atom of the phenanthroline. As the complexes under study **1** and **2** are isomorphous to **3**, we expect to adopt similar geometries.

### 3.2. Characterization of [ReOC*l*_3_(*α*-diimine)] Complexes

#### 3.2.1. Infrared Data

The IR spectra of **1**, **2**, and **3 ** exhibit very strong bands at 939, 988, and 985 cm^−1^, respectively, assignable to the **ν**(Re=O) mode. These values compare well with those reported for rhenium (V) complexes containing a chelating ligand in their coordination sphere [32]. The characteristic bands corresponding to the **ν**(CN) and **ν**(C=C) modes of the quinoxaline and diimine ligand appear in the range 1680–1420 cm^−1^ in the IR spectra of the complexes **1**–**3** [20, 25, 32].

#### 3.2.2. NMR Spectroscopy

The oxorhenium complexes **1**–**3** give well-defined ^1^H-NMR spectra, which permit unambiguous identification and assessment of purity. The proton chemical shifts are assigned with the aid of ^1^H-COSY experiments (provided in Section 2).

**Figure 2:**
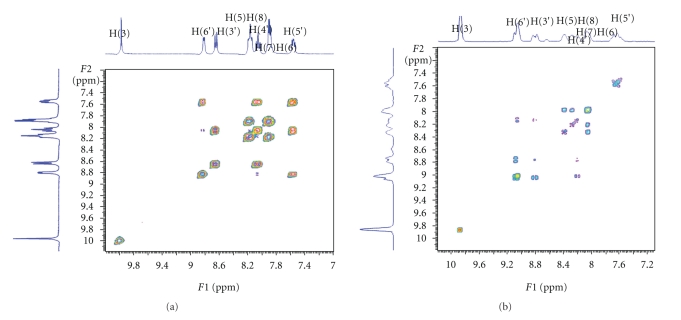
COSY-NMR spectra of pq (a) and [ReOCl_3_pq] (b) in methanol.

It is worth to discuss the complexation of 2-(2′ pyridyl)quinoxaline to the [ReOCl_3_(PPh_3_)_2_] moiety since it entails two major changes: the first involves the configuration of 2-2′pq switching from the *syn/cis*- to the *anti/trans*-conformation, while the second is related to the withdrawal of its electron density by the metal. These changes are reflected on the NMR spectra of the complex. Figure 2 displays the ^1^H–^1^H COSY spectra of the free ligand L and of the complex, which are important for assigning of the 1D-NMR peaks.

The NMR spectrum of the coordinated ligand L differs from that of the free ligand (Figure 2) mainly due to the separation of H_5_ and H_8_ signals and to an overall downfield shift. The latter is due to the reducing electron charge of the protons after coordination of the ligand. The most downfield shifted peak remains the H_3_(s) [31]. The same switching from *syn/cis*- to the *anti/trans*-conformation is also observed in the spectrum due to the complexation of 2-2′-bipyridine ligand.

#### 3.2.3. UV-Vis Spectroscopy

The electronic absorption spectra of [ReOCl_3_(*α*-diimine)] in the area between 300 and 800 nm are shown in Table 1. The UV-Vis spectra of **1**–**3 **exhibit few intense bands in the range of 570–200 nm and a weak absorption in the low range 730–750 nm. According to TD-DFT calculations [32], the longest wavelength experimental band of **1**, **2**, and **3** at 732, 750, and 739 nm, respectively, is attributed to the transition of d → d character. The broad MLCT absorption band appears at 571, 480, and 459 nm for complexes **1**, **2**, and **3**, respectively. It originates from the d_*Re*_ orbitals to the *π*_*α*−diimine_* orbitals [32] and it is blue shifted in less polar solvents (Table 1), indicating its charge transfer character. The higher energy absorption bands at UV region are attributed to ligand-ligand charge transfer transitions, namely, (*π*)Cl/(*π*)O/(*π*)diimine → π*(diimine), interligand transitions.

#### 3.2.4. Electrochemical Behaviour of Complexes

 The redox properties of the compounds 1–3 have been investigated by cyclic voltammetry, at a Pt electrode, usually in a 0.1 M TBABF_4_-DMF solution at 25 ± 0.2°C, and the measured redox potentials (in V *versus* SCE) are given in Table 1. Solutions were deoxygenated by purging with argon gas for 15 minutes prior to the measurements; during the measurements a stream of argon was passed over the solution. All the studied complexes exhibit (Table 1) a reduction wave which is usually followed, at the lowest potential, by a second one. These waves often correspond to single-electron reversible processes, being assigned to the d^n^ → d^n+1^  and d^n+1^ → d^n+2^ metal reductions. Previous studies have shown that the diimine ligand influences the reduction potential of the compounds. The oxidation for all three complexes occurs at very similar potentials. Both reduction potentials occur at negative potentials.

### 3.3. Biological Assays

#### 3.3.1. T_m_ Measurements

**Figure 3:**
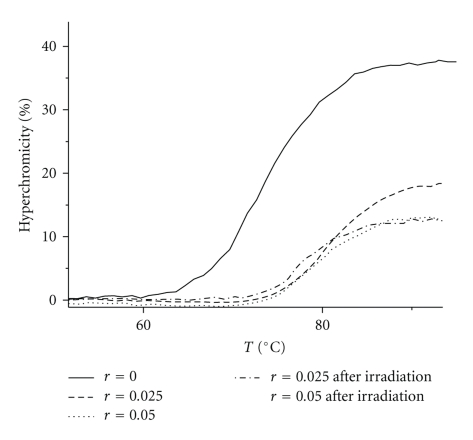
Thermal denaturation curves of C.T.-DNA in the presence of the complex ReOCl_3_pq before and after irradiation at increasing molar ratios *r*, pH = 7.0.

The study of the melting curves of C.T.-DNA indicates that the interaction of DNA with the oxorhenium (V) complexes **1** and **2**, leads to the stabilization of the double helix in proportion to the ratio *r* = [ReOCl_3_(*α*-diimine)]/[CT-DNA] (Table 2). The stabilization can be assumed by the raising of the *T*_*m*_ which is finally observed in analogy with *r*, reaching at +10.85°C for **1** at *r* = 0.05 and pH = 7.0 (Figure 3). In the presence of all three complexes (**1**–**3**) the thermal stabilization of CT-DNA is observed in the series 1 > 2 > 3. Moreover, the reduction of the final hyperchromicity and the decrease of the slope of melting curves (increase of the transition) provide a strong evidence of the interaction which leads to DNA helix stabilization [33]. “Premelting effects” of the double helix which could be caused by the binding of oxorhenium (V) complexes, probably via allosteric effects, are rather not taking place since they would result in DNA helix destabilization [34]. The reversibility measurements of the **1** and **3** binding to CT-DNA (cooling the samples and reheating them) showed completely super-imposable results onto the first heating scans. This suggests that the species present in the solution of **1** or **3**, do not alone inhibit reannealing, associating irreversibly with the single strand which is similar to the results obtained for the free ligands. Although the complex [ReOCl_3_phen] is isostructural to the complexes [ReOCl_3_pq] and [ReOCl_3_bpy], it presents different behaviour when interacts with CT-DNA. In both buffered pH 7.0 and pH 5.0 solutions, at all ratios, the melting points of CT-DNA are almost the same with free CT-DNA. Identical results have been obtained when CT-DNA was treated with all free ligands under investigation. So we can conclude that there is almost no interaction between the complex [ReOCl_3_phen] and CT DNA. No observable changes are measured after irradiation.

#### 3.3.2. CV Studies

**Figure 4:**
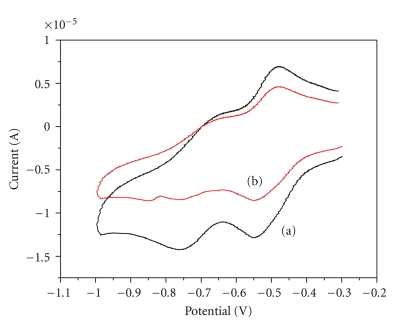
Cyclic voltagramm of **1 (**0.1 mM). (a) in the absence and (b) in the presence of DNA. Supporting electrolyte 0.1 mM TBABF_4_ in DMF.

Electrochemical investigation of complex-DNA interactions can provide a useful complement to other methods and yield information about the mechanism of the interaction [23, 24, 35]. Figure 4 shows the cyclic voltammograms of the complex at the absence and the presence of DNA. A new irreversible redox peak appeared after the addition of CT DNA to complex **1**, whereas the intensity of all peaks decreased significantly, suggesting the existence of an interaction between **1** and CT DNA. The observed decrease in current can be attributed both to the intercalation of complex **1** into the base pairs of DNA by the planar pq ligand [23, 24] and to an equilibrium mixture of free and DNA-bound complex to the electrode surface [35]. The experimental results are different for **2**, where the current intensity of all the peaks decreased significantly after addition of CT DNA, and for **3** where no changes are observed in its cyclic voltammogram implying that complex **3** does not interact with DNA.

#### 3.3.3. Viscosity Measurements

**Figure 5:**
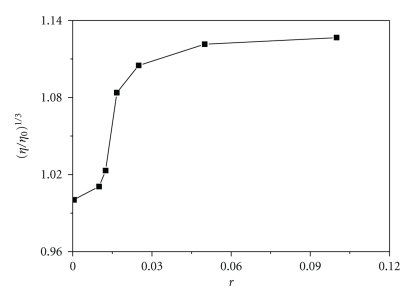
Effect of increasing amounts of complex 1 (0–100 *μ*M) on the relative viscosity of CT-DNA (100 *μ*M).

Optical photophysical probe provides necessary but not sufficient clues to support binding modes [24, 36], whereas hydrodynamic measurements that are sensitive to the length change are regarded as the most critical tests of a binding model in solution. Thus, to further clarify the interaction between 1 and DNA, we carried out viscosity measurements. Because the latter are ionic-strength-and-concentration dependent, we used a buffer of the same ionic strength for all measurements. Figure 5 shows the relative viscosity of DNA (100 lM) in the presence of varying amounts of complex **1**. As we observe, the relative viscosity increases from 0.98 to 1.14 in relative low ratios, [complex]/[DNA], while it remains almost constant there after. The observed increase is at the same extent with the one observed when an intercalator is used, for example ethidium bromide—a well known intercalator, at the same ratios as complex **1** [24, 37]. This behaviour suggests that complex **1** intercalates to CT-DNA in accordance with the previous results.

#### 3.3.4. DNA Photocleavage

**Figure 6:**
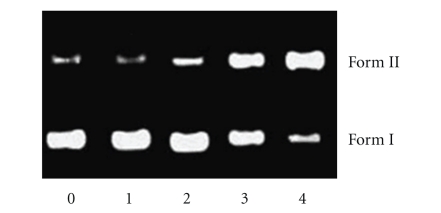
Photoactivated cleavage of pBR 322 DNA in the presence of Re(V) complex, light after 60 minutes irradiation at *λ* > 400 nm. Lane 0, DNA alone; lanes 1–4, in the different concentrations of complex **1**: (1) 0; (2) 20; (3) 40; (4) 60 lM.

**Figure 7:**
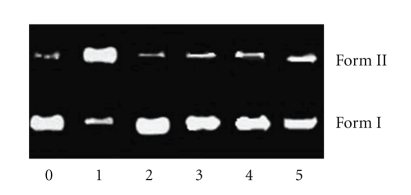
Photoactivated cleavage of pBR 322 DNA in the presence of 20 *μ*M of complex **1** and different inhibitors after irradiation at *λ* > 400 nm for 60 minutes. Lane 0, DNA control; lane 1, no inhibitor; lanes 2–6: (2) histidine (5 mM), (3) DMSO (0.2 M), (4) ethanol (0.2 M), (5) sodium formate (5 mM).

The ability of complex **1** to cleave DNA upon irradiation was determined by agarose gel electrophoresis [38]. When circular plasmid DNA in the presence of an inorganic molecule is subject to electrophoresis, relatively fast migration will be observed for the intact supercoil form (Form I). If scission occurs on one strand (nicking), the supercoil will relax to generate a slower-moving open circular form (Form II). If both strands are cleaved, a linear form (Form III) that migrates between Forms I and II will be generated [38]. Figure 6 shows gel electrophoresis separation of pBR 322 DNA after incubation with complex **1** and irradiation by visible light (*λ* > 400 nm) for 1 hour. Complex **1** exhibited concentration-dependent single-strand cleavage of supercoiled Form I into nicked Form II. Control experiment (lane 0) suggests that untreated DNA does not show any cleavage upon irradiation, with increasing concentration of **1** (lanes 1–3); the amount of Form I of pBR322 DNA diminishes gradually, whereas that of Form II increases. Under comparable experimental conditions, complex **2** exhibits more effective DNA cleavage activity than complex **1**. In order to identify the nature of the reactive species that are responsible for the photo-activated cleavage of the plasmid DNA, we further investigated the influence of different potentially inhibiting agents. In the case of complex **1** (Figure 7), studies with the single oxygen quencher histidine were carried out and the plasmid cleavage was inhibited (lane 2), which confirmed that the singlet oxygen was involved in the cleavage. At the same time, in the presence of different hydroxyl radical scavengers such as DMSO (lane 3), ethanol (lane 4) and sodium formate (lane 5), different degrees of inhibition in the photo-induced cleavage of the plasmid by complex **1** were also observed. This indicates that hydroxyl radical also plays a significant role in the photocleavage mechanism for **1**, and the photoreduction of ReO complexes with concomitant hydroxide oxidation is an important step in the DNA cleavage reaction [38].

## 4. Conclusions

In summary, a new oxorhenium (V) complex namely, [ReOCl_3_pq] (**1**) together with its isostructural [ReOCl_3_bpy] (**2**) and [ReOCl_3_phen] (**3**) has been synthesized and characterized by elemental analyses, FT-IR, UV-Vis spectra, 1D, and 2D NMR, ESI-MS, and CV. The interactions between complexes **1**–**3** (as well as all a-diimine ligands) and calf thymus DNA have been investigated using UV-spectra, thermal denaturation measurements, CV, and viscosity measurements. Furthermore, the photocleavage of the plasmid pBR 322 DNA has been investigated by agarose gel electrophoresis. Remarkably, our results reveal that both complexes **1** and **2** bind to DNA by intercalation, with the planar diimine (pq or bpy) ligands stacked between the base pairs of the DNA. Complex **1** can efficiently cleave the plasmid pBR 322 DNA, upon irradiation whereas the reactive species responsible for its cleavage are the singlet oxygen and the hydroxyl radical. The thermal stabilization of DNA is reversed to the promoted photocleavage at physiological pH and temperature, indicating the differences in interactions between the base pairs of DNA and the ground or the excited state of the complexes. Similar observations have also been reported for rhenium (I) complexes [39, 40]. These complexes may be useful to probe nucleic acid structures and in the development of DNA agents. More detailed photophysical and biophysical studies designed to address the nature of the interactions of oxorhenium (V) are underway.

## Figures and Tables

**Table 1:** Electrochemical and absorption data of the oxorhenium (V) complexes.

Complex	E_1/2_/V versus SCE^a^		*λ*_max _/nm (*ε*/M^−1^cm^−1^)^b^	*λ*_max _/nm (*ε*/M^−1^cm^−1^)^c^
	E_pa_ (V)	E_pc_ (V)		
[ReOCl_3_pq] (**1**)	−0.52	−0.70	268.5 (4835)	272.4 (4820)
			571.0 (3330)	581.2 (3520)
			732.1 (1227)	738.3 (1337)

[ReOCl_3_bpy] (**2**)	−0.54	−0.72	295.3 (4315)	294.1 (4330)
			480.8 (3430)	493.0 (3520)
			750.2 (1450)	753.0 (1480)

[ReOCl_3_phen] (**3**)	−0.44	−0.61	270.6 (4580)	268.3 (4350)
			459.6 (3200)	463.7 (3300)
			739.6 (1100)	745.0 (1150)

^a^All complexes were measured in 0.1 M TBABF_4_-DMF; error in potential was ±0.01 V; *T* = 25 ± 0.2°C; scan rate = 100 mV · s^−1^

^b^In  CH_3_OH, [C] = 10^−2^ M

^c^In  CH_3_COCH_3_, [C] = 10^−2^ M.

**Table 2:** Melting temperatures of CT-DNA in 10 mM buffer (pH 7.0 and pH 5.0) in the presence of complexes 1–3, before and after irradiation (*λ* = 420–1000 nm).

C.T.-DNA +^a^		R = 0.025			R = 0.050			R = 0.025*			R = 0.050*		
	pH	*T*_*m*_°C	Δ*T*_*m*_	%hyp	*T*_*m*_°C	Δ*T*_*m*_	%hyp	*T*_*m*_°C	Δ*T*_*m*_	%hyp	*T*_*m*_°C	Δ*T*_*m*_	%hyp
[ReOCl_3_pq]	7.0	80.1	+9.2	18.0	81.8	+10.9	13.0	77.9	+7.0	12.0	79.1	+9.0	12.0
**(1)**	5.0	81.1	+8.0	27.0	80.2	+7.1	19.0	78.1	+5.0	28.0	79.1	+6.0	19.0

[ReOCl_3_bpy]	7.0	76.9	+6.0	44.0	76.9	+6.0	39.0	75.4	+4.5	45.0	75.1	+5.0	36.0
**(2)**	5.0	76.1	+3.0	20.0	77.1	+4.0	20.0	73.1	+0.0	20.0	76.1	+3.0	20.0

[ReOCl_3_phen]	7.0	71.1	+0.2		71.4	+0.5		70.9	+0.0		74.2	+0.3	
**(4)**	5.0	73.1	+0.0		73.3	+0.2		73.1	−0.0		73.1	+0.0	

*Illuminated samples. ^a^The melting point of the free CT-DNA is 70.9°C (pH 7.0) and 73.1°C (pH 5.0).
